# Real-time nonlinear parameter estimation and tracking control of unmanned aerial vehicles in closed-loop

**DOI:** 10.1038/s41598-023-29544-6

**Published:** 2023-02-22

**Authors:** Imil Hamda Imran, Aydin Can, Rustam Stolkin, Allahyar Montazeri

**Affiliations:** 1 Department of Engineering, Lancaster University, Bailrigg, Lancaster, LA1 4YW UK; 2 Applied Research Center for Metrology, Standards and Testing, King Fahd University of Petroleum and Minerals, Dhahran, 31261 Saudi Arabia; 3 Extreme Robotics Lab (ERL), School of Metallurgy and Materials, University of Birmingham, Edgbaston, Birmingham, B15 2TT UK

**Keywords:** Engineering, Aerospace engineering, Electrical and electronic engineering

## Abstract

The real-time unknown parameter estimation and adaptive tracking control problems are investigated in this paper for a six degrees of freedom (6-DOF) of under-actuated quadrotor unmanned aerial vehicle (UAV). A virtual proportional derivative (PD) controller is designed to maintain the translational dynamics. Two adaptive schemes are developed to handle the attitude dynamics of the UAV with several unknown parameters. In the beginning, a classical adaptive scheme (CAS) using the certainty equivalence principle is proposed and designed. The idea is to design a controller for an ideal situation by assuming the unknown parameters were known. Then the unknown parameters are replaced by their estimation. A theoretical analysis is provided to ensure the trajectory tracking of the adaptive controller. However, an inherent drawback of this scheme is that there is no guarantee for the estimated parameters to converge to the actual values. To address this issue, a new adaptive scheme (NAS) is developed as the next step by adding a continuously differentiable function to the control structure. The proposed technique guarantees handling of the parametric uncertainties with an appropriate design manifold. A rigorous analytical proof, numerical simulation analyses, and experimental validation are presented to show the effectiveness of the proposed control design.

## Introduction

Over the past decade, research and development on quadrotor UAV have drawn the attention of various researchers and industries. Quadrotor deployment has many potential benefits as compared to the traditional methods carried out by a human. Its deployment is specially useful for missions which are risky for humans. Further to improving the safety in executing tasks, it can also enhance the efficiency by saving the cost and time. Several UAV applications can be found in nuclear decommissioning, data collection, volcano monitoring, geographical photography, and creative industries^1–3^. From the control engineers’ perspective, one of the hottest research topics for autonomous operation and cognition of UAVs is to operate a single UAV or multiple UAVs interacting with other robots and sensors as a cyber-physical system^4,5^. Many control approaches have been investigated under various settings^5^. The main objective in designing control systems for UAVs, is to make them more autonomous and less dependent to the operator while enabling the UAVs to have harsh maneuvers by compensating the nonlinearity and various sources of uncertainties the UAV might experience in realistic situations.

UAV is an under-actuated nonlinear system, with four individual rotors to maintain a highly coupled six states as the system output. The rotors can be installed in a plus or cross configuration. The quadrotor has three states related to translational motion allowing UAV to move in the backward, forward, lateral, and vertical directions. The rest of the dynamics are related to the rotational or attitude motions, namely referred to as roll, pitch and yaw angles. The main concern in the trajectory tracking problem is to design the controller for the rotational dynamics. This is due to the natural behavior of UAV as an under-actuated system, where the position tracking control problem is maintained by controlling its attitude or rotational dynamics.

## Related works and main contributions

The presence of nonlinearities in the system dynamics is one of the essential issues in designing the controller for the attitude dynamics. A proper and suitable nonlinear controller has an important role in maintaining the UAV motions with a full nonlinear behavior. Several studies have been presented to address the trajectory tracking problem, especially for the attitude dynamics of UAVs. One of the common approaches is the feedback linearization method as developed in^6,7^.

However, the parameters of the UAV are not always available or will change over time for the feedback control design in various practical situations. These unknown parameters may cause more complicated technical challenges in designing a proper controller. In general, there are two major research lines to tackle this issue. The first research direction is dealing with robust controllers. The idea behind this approach is to propose a feedback controller to dominate the uncertainties in the system dynamics. In this way, the controller guarantees to handle the uncertainties within a particular bound. This disadvantage of this approach is that the bound of the uncertainties should be available a priori to ensure the stability of the closed-loop control system. One of the popular methods in this research line is the sliding mode control (SMC). SMC is widely implemented in various practical situations as it is less sensitive to parametric uncertainties and disturbances as presented in^8–10^. However, chattering is a common issue in designing SMC for the autonomous system. Several studies have been investigated to reduce the chattering as well as to compensate the effect of unmodelled dynamics as presented in^11–13^.

The second major research line is adaptive control method. This approach is useful to handle the unknown constant parameters in the system dynamics. The traditional approach or CAS was initially proposed in the literature using the certainty equivalence principle. The idea behind this approach is to cancel the nonlinear terms containing unknown constant parameters. This scheme suggests a two-step control design procedure to handle the uncertainties. A controller under an ideal condition is designed in the first step, where all parameters are assumed to be known for feedback controller. In the second step, every unknown constant parameter is replaced by its estimation generated by an adaptive law along the gradient of Lyapunov function. The perfect cancellation of the nonlinear term is deemed to be achieved by driving the estimated parameter to converge to the actual value. This technique has a simple structure for practical implementation; however, it contains an inherent drawback. In this technique, there is no guarantee that the parameter estimation error converges to zero as it fully relies on the system dynamic states. The estimated parameters are updated constantly (even though they reach their actual values) as long as the states of system dynamics don’t reach the equilibrium point. Nonetheless, the parameter estimation adaptive law will stop the update as soon as the system dynamics states reach the equilibrium point. For some more information on this technique, an interested reader may refer to in^5,14,15^.

Another adaptive technique to deal with uncertain parameters is the model reference adaptive control (MRAC). This technique also works using the certainty equivalence principle. A reference model is added to the control structure to estimate the unknown constant parameters as presented in^16,17^. However, this method has a major drawback to guarantee the stability of the closed-loop system as investigated in^18^. To rectify this problem, $L_1$ adaptive control scheme was proposed by adding a linear filter in the whole control structure^19^. This technique was studied further in^20^ for the collaborative UAVs. Note that this scheme doesn’t guarantee the estimate parameters to converge to the actual value of the unknown parameters as it was tuned for the worst-case condition. Application of this scheme in UAVs can be found in^21^. The mismatch between the estimate parameters and the actual values are used to update the parameters of the system. As a result, this approach can be implemented mainly in limited cases.

Model predictive control (MPC) is one of the common methods proposed in the literature to handle the uncertainties. For example, adaptive MPC with extended state observer was studied in^22^ for UAVs in a networked setting. The unknown disturbances and system uncertainties were handled successfully. However, the controller was not designed to estimate the unknown parameters in the class of the nonlinear function. The difficulties in estimating the unknown parameters of a nonlinear function in closed-loop has been avoided by linearizing the system dynamic in this method. It is not surprising as this is an essential assumption in designing a controller using the linear MPC technique. Another result in which MPC is used to handle the disturbances and sensor faults is reported in^23^.

Another common technique used to handle the uncertainties is the intelligent computation approach. For example, neural network (NN)^24,25^ was designed for autonomous agents in a networked setting and genetic algorithm (GA)^26^ is proposed to address the uncertainties in a robotic manipulator. However, this approach has two major issues. The first issue is that both the tracking error and the parameter estimation error are not converging to zero asymptotically and will have a residual error. This is caused by the mismatch between the approximator and the unknown nonlinear function. The second issue is that the intelligent computation requires a high performance embedded computer in many cases to estimate the uncertainties. As a result, this approach can only be implemented in limited practical scenarios.

Immersion and invariance (I &I) was developed to address the open problem in CAS as studied in^27,28^. This technique also has a two-step control design. In the first step, the system was designed to satisfy the input to state stability (ISS) condition, where the nonlinear term with unknown parameters are assumed to be zero. Then the unknown nonlinear terms are taken into account in the second step. The adaptive term is designed by replacing the unknown parameter with its estimation and an additional appropriate continuous function design to assist the parameter estimation algorithm to converge in the right direction. In this situation, stability can be guaranteed by driving the mismatch estimation error to a specific manifold. Note that the stability condition is derived by looking at the error dynamics for the parameter estimation rather than the closed-loop system dynamics. Hence, it is reasonable to apply the proposed controller under the ISS condition.

Application of I &I adaptive to handle the unknown parameters in UAVs can be found in^21,29^. However, the I &I adaptive law has the major drawback of requiring the mismatch error between the actual value and the estimated parameters. As a result, the technique contains a significant bottleneck to be implemented in practical situations. To solve this issue, we develop two adaptive schemes for a quadrotor UAV with unknown constant parameters in this study. A traditional or CAS is designed in the first scheme to handle the unknown parameters using the certainty equivalence principle. This scheme suggests two steps to estimate the unknown parameters of the system dynamic. First, the controller is designed for the system under the ideal condition by assuming all the system parameters to be available for the feedback control design. Then the unknown parameters are replaced by their estimated values updated by adaptation law in the second step. As mentioned above, this classical scheme contains an inherent drawback to estimate the unknown parameters.

Inspired by the studies in^27,30,31^, we develop NAS in the second scheme to handle multiple uncertain parameters in the rotational dynamics of the UAV. An appropriate manifold is designed to eliminate the effect of unknown parameters, where it is not necessary to drive the steady-state parametric estimation error to zero, but it follows a pre-defined manifold of UAV states. Here the ISS condition is relaxed compared to the result in^27^. The stability analysis is presented on both the closed-loop trajectory tracking and parameter estimation error dynamics. The mismatch between the actual values and estimate parameters is not required for both feedback control design and adaptation law. In this way, we can remove the inherent drawback in CAS. This extension is a very important extension for the current approach to deal with unknown constant parameters in practical implementation. A virtual PD controller is designed to track the desired position in the outer loop of the proposed nested control structure. The major contributions of this study are summarized as follows:

- The asymptotic stablisation and trajectory tracking problem for UAVs as a fully coupled and nonlinear underactuated system with unknown parameters is studied using adaptive control schemes.
- A new adaptive control scheme (NAS) for a UAV with unknown parameters is proposed to estimate the unknown parameters of the UAV in closed-loop so that it guarantees the estimated parameters of the UAV converge to a known value from which the value of true parameters can be derived. This is an extension compared to the classical scheme (CAS) in which there is no guarantee that the parameter estimation error converges to zero.
- Through extensive numerical simulations the superiority of NAS are evaluated and compared with various existing methods and implemented on a real UAV in an experimental setting.
- Some simulations and practical implementations for mini-drone are conducted to verify the performance of our scheme. Especially in the practical test, we demonstrate that our new scheme has a simple structure that makes it possible to be tested in a mini-drone with limited hardware computation.

The remainder of this paper is organized as follows. A dynamic model of UAV is presented in “System dynamics of UAV” section. Following that, the position tracking control for the outer loop and the two proposed adaptive schemes for the attitude dynamics are presented in “Proposed control design” section. Then in “Simulation and experimental results” section, with extensive numerical analyses and simulation results the efficacy of the proposed controllers are demonstrated. Finally, the paper is concluded in “Conclusion and directions for future work” section with a conclusion and some suggestions for future work.

## System dynamics of UAV

The 6-DOF dynamic model of the quadrotor UAV is represented by the following states

$$\begin{aligned} \eta = \begin{bmatrix} \eta _1 \\ \eta _2 \end{bmatrix}, \; \nu = \begin{bmatrix} \nu _1 \\ \nu _2 \end{bmatrix} , \end{aligned}$$

where $$\eta _1 = \begin{bmatrix} x&y&z \end{bmatrix}^{\tiny \textsf {T}}\in \mathbb {R}^{3\times 1}$$ is the absolute position of UAV composed of forward (*x*), lateral (*y*) and vertical (*z*) motions; $$\eta _2 = \begin{bmatrix} \phi&\theta&\psi \end{bmatrix}^{\tiny \textsf {T}}\in \mathbb {R}^{3\times 1}$$ is the attitude composed by three Euler angles i.e. roll ($\phi$), pitch ($\theta$) and yaw ($\psi$) motions; $$\nu _1 = \begin{bmatrix} u&v&w \end{bmatrix}^{\tiny \textsf {T}}\in \mathbb {R}^{3\times 1}$$ is a linear velocity; and $$\nu _2 = \begin{bmatrix} p&q&r \end{bmatrix}^{\tiny \textsf {T}}\in \mathbb {R}^{3\times 1}$$ is an angular velocity. The dynamic of the UAV can be derived as a result of the coupling between the inertial and body frames. This coupling can be expressed by the transformation matrices $J_1(\eta _2)$ and $J_2(\eta _2)$

$$\begin{aligned} J_1(\eta _2)&= \left[ \begin{matrix} \cos \theta \cos \psi &{} \sin \phi \sin \theta \cos \psi - \cos \phi \sin \psi &{} \cos \phi \sin \theta \cos \psi + \sin \phi \sin \psi \\ \cos \theta \sin \psi &{} \sin \phi \sin \theta \sin \psi + \cos \phi \cos \psi &{} \cos \phi \sin \theta \sin \psi - \sin \phi \cos \psi \\ -\sin \theta &{} \sin \phi \cos \theta &{} \cos \phi \cos \theta \end{matrix}\right] \\ J_2(\eta _2)&= \begin{bmatrix} 1 &{} \sin \phi \tan \theta &{} \cos \phi \tan \theta \\ 0 &{} \cos \phi &{} -\sin \phi \\ 0 &{} \frac{\cos \phi }{\cos \theta } &{} \frac{\cos \phi }{\cos \theta } \end{bmatrix} , \end{aligned}$$

Both $\phi$ and $\theta$ are assumed between $-\frac{\pi }{2}$ to $\frac{\pi }{2}$. As results, $\cos \phi$ and $\cos \theta$ are non-zero, hence $J_{1}^{^{\tiny \textsf {T}}}(\eta _2) = J_{1}^{-1}(\eta _2)$. The dynamic coupling between position and orientation vectors can be represented by the following transformation

1 $$\begin{aligned} \dot{\eta }_1 = J_1(\eta _2)\nu _1 , \; \dot{\eta }_2 = J_2(\eta _2)\nu _2 . \end{aligned}$$

**Figure 1:**
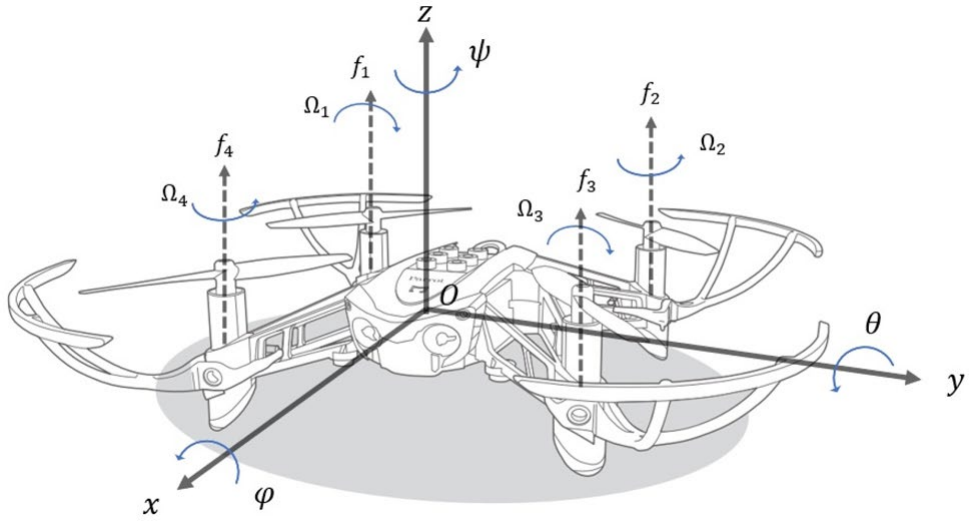
Body fixed and earth reference frame of a 6-DOF UAV.

The use of both body fixed frame and the inertial (earth) coordinates are required to study 6-DOF motions of UAV. Both position and orientation are described with respect to the inertial frame. In another side, both linear and angular velocities are described with respect to the body frame. The illustration of these two coordinate frames can be seen in Fig. 1.

As derived in^21^, the translational dynamic of the quadrotor UAV can be represented as

2 $$\begin{aligned} \ddot{\eta }_1 = -g z_e + J_1(\eta _2) \frac{u}{m} z_e - \frac{k_t}{m} \dot{\eta }_1 , \end{aligned}$$

where $g \in \mathbb {R}$, $u \in \mathbb {R}$, $m \in \mathbb {R}$, and $k_t \in \mathbb {R}$ and are gravity acceleration, thrust force, mass, and translational drag coefficient, respectively. Vector $$z_e = \begin{bmatrix} 0&0&1 \end{bmatrix}^{\tiny \textsf {T}}\in \mathbb {R}^{3\times 1}$$ is the unitary vector in *z* direction. The attitude dynamics is expressed by

3 $$\begin{aligned} \dot{\nu }_2 = I_{M}^{-1} ( -(\nu _2 \times I_M \nu _2) - I_R (\nu _2 \times z_e) \Omega - k_r \nu _2 + \tau ) , \end{aligned}$$

where $I_R \in \mathbb {R}$ and $k_r \in \mathbb {R}$ are propeller inertia and rotational drag coefficient, respectively. Vector $$\tau = \begin{bmatrix} \tau _p&\tau _q&\tau _r \end{bmatrix}^{\tiny \textsf {T}}\in \mathbb {R}^{3\times 1}$$ shows the torques acting on the body frame in roll, pitch, and yaw directions. The matrix $$I_M = \text {diag} \begin{bmatrix} I_x&I_y&I_z \end{bmatrix} \in \mathbb {R}^{3\times 3}$$ is a diagonal inertia matrix. The relative angular speed $\Omega \in \mathbb {R}$ generated by the motors is

4 $$\begin{aligned} \Omega = \Omega _1 - \Omega _2 + \Omega _3 - \Omega _4 . \end{aligned}$$

The thrust force *u*, generated by each propeller can be calculated as

5 $$\begin{aligned} u = \sum _{\iota =1}^{4} f_\iota = \sum _{\iota =1}^{4} k_\iota \Omega _\iota ^2, \end{aligned}$$

where $f_\iota \in \mathbb {R}$ is the upward-lifting force generated by every rotor and $k_\iota \in \mathbb {R}$ is a positive constant. From (2) and (3), it can be seen that the number of control inputs is less than the number of degrees of freedom, and hence the UAV is a nonlinear under-actuated system.

The UAV considered in this paper has a cross configuration. The propeller forces and the input control signals acting on the UAV body are related according to (6)

6 $$\begin{aligned} \begin{bmatrix} u \\ \tau _p \\ \tau _q \\ \tau _r \end{bmatrix} = \begin{bmatrix} 1 &{} l &{} 1 &{} 1 \\ l\frac{\sqrt{(}2)}{2} &{} -l\frac{\sqrt{(}2)}{2} &{} -l\frac{\sqrt{(}2)}{2} &{} l\frac{\sqrt{(}2)}{2} \\ l\frac{\sqrt{(}2)}{2} &{} l\frac{\sqrt{(}2)}{2} &{} -l\frac{\sqrt{(}2)}{2} &{} -l\frac{\sqrt{(}2)}{2} \\ d &{} -d &{} d &{} -d \end{bmatrix} \begin{bmatrix} f_1 \\ f_2 \\ f_3 \\ f_4 \end{bmatrix} , \end{aligned}$$

where $l \in \mathbb {R}$ is the arm length and $d \in \mathbb {R}$ is the drag factor.

The attitude dynamic in (3) with an additional external disturbance can be rewritten in the linearly parameterized form as

7 $$\begin{aligned} \dot{\nu }_2 = f(\nu _2) + g_1(\nu _2)I_R + g_2(\nu _2)k_r + h \delta + I_{M}^{-1} \tau , \end{aligned}$$

where

$$\begin{aligned} f(\nu _2) = \begin{bmatrix} \frac{I_y - I_z}{I_x} qr \\ \frac{I_z - I_x}{I_y} pr \\ \frac{I_x - I_y}{I_z} pq \end{bmatrix}, \; g_1(\nu _2) = \begin{bmatrix} \frac{-\Omega q }{I_x} \\ \frac{\Omega p}{I_y} \\ 0 \end{bmatrix}, \; g_2(\nu _2) = -\begin{bmatrix} \frac{p}{I_x} \\ \frac{q}{I_y} \\ \frac{r}{I_z} \end{bmatrix}. \end{aligned}$$

In (7), $I_R$ and $k_r$ are unknown parameters to be estimated for the feedback control design. Also, $h \delta$ is an external disturbance acting on the body frame, in which *h* is a known vector as a function of time and $\delta$ is an unknown scalar. The full dynamic equations of the UAV can be represented by looking at Eqs. (1), (2) and (7) together. As a nonlinear function of the UAV states is present in all equations and the translational and rotational motions are coupled together with these three equations, the nonlinear fully coupled nature of the dynamic equations is exploited for the control design.

## Proposed control design

As discussed in the previous section, the quadrotor UAV is an under-actuated system, in which the six system states should be controlled by four control inputs. There is also a strong coupling between the translational and attitude dynamic of UAV. In this section, a nested control strategy for trajectory tracking of the quadrotor dynamic is proposed. The position tracking error is controlled by a PD controller in the outer loop. However, two adaptive control schemes are proposed to control the attitude dynamic in the presence of uncertain parameters of the system. As the first step, CAS is designed using the certainty equivalence principle. In this technique, the adaptive law is designed to force the parameter estimation error to converge to zero. Nevertheless, in the second technique, NAS is proposed by adding a certain continuous differentiable function in the structure of the adaptive control design. Contrary to CAS, here the estimation error is converging to a manifold rather than zero.

### Translational control design

The tracking controller for the translational dynamic is designed by defining the tracking error of the system as

8 $$\begin{aligned} \tilde{\eta }_1 = \eta _{1_d} - \eta _1 , \end{aligned}$$

where $\tilde{\eta }_1$ and $\eta _{1_d}$ are the error vector position and the desired vector position, respectively. The double integrator dynamics of (8) can be written as

9 $$\begin{aligned} \ddot{\tilde{\eta }}_1 + K_D \dot{\tilde{\eta }}_1 + K_P \tilde{\eta }_1 = 0, \end{aligned}$$

where $K_P \in \mathbb {R}^{3\times 3}$ and $K_D \in \mathbb {R}^{3\times 3}$ are the diagonal matrices denoting the gains for the proportional and derivative terms, respectively. By selecting $K_P$ and $K_D$ to be positive definite matrices, the system dynamics (9) satisfies the Routh-Hurwitz stability criterion by having $\lim _{t\rightarrow \infty } \tilde{\eta }_1 (t) = 0$. The dynamics in (8) can be rewritten as

10 $$\begin{aligned} \ddot{\eta }_1 = \ddot{\eta }_{1_d} + K_D (\dot{\eta }_{1_d} - \dot{\eta }_1) + K_P (\eta _{1_d} - \eta _1). \end{aligned}$$

Inspired by^32^,the virtual control input $$U=\ddot{\eta }_1=\begin{bmatrix} U_1&U_2&U_3 \end{bmatrix}^{\tiny \textsf {T}}$$ is defined and substituted in (2). This yields

11 $$\begin{aligned} U = -g z_e + J_1(\eta _2) \frac{u}{m} z_e - \frac{k_t}{m} \dot{\eta }_1 , \end{aligned}$$

or

12 $$\begin{aligned} \frac{u}{m} z_e = J_1^{-1} (\eta _2)\left( U + g z_e + \frac{k_t}{m} \dot{\eta }_1\right) , \end{aligned}$$

By expanding (12), it would be straightforward to verify that

13 $$\begin{aligned}{} & {} \left( U_1 + \frac{k_t}{m}\dot{x}\right) \cos \theta \cos \psi + \left( U_2 + \frac{k_t}{m}\dot{y}\right) \cos \theta \sin \psi - \left( U_3+g+\frac{k_t}{m} \dot{z}\right) \sin \theta = 0 , \end{aligned}$$

14 $$\begin{aligned}{} & {} \left( U_1 + \frac{k_t}{m}\dot{x}\right) (\sin \phi \sin \theta \cos \psi - \cos \phi \sin \psi ) + \left( U_2 + \frac{k_t}{m}\dot{y}\right) (\sin \phi \sin \theta \sin \psi + \cos \phi \cos \psi ) \nonumber \\{} & {} \qquad + \left( U_3+g+\frac{k_t}{m}\dot{z}\right) \sin \phi \cos \theta = 0 , \end{aligned}$$

15 $$\begin{aligned}{} & {} \left( U_1 + \frac{k_t}{m}\dot{x}\right) (\cos \phi \sin \theta \cos \psi + \sin \phi \sin \psi ) + \left( U_2 + \frac{k_t}{m}\dot{y}\right) (\cos \phi \sin \theta \sin \psi - \sin \phi \cos \psi ) \nonumber \\{} & {} \qquad + \left( U_3+g+\frac{k_t}{m}\dot{z}\right) \cos \phi \cos \theta = \frac{u}{m} . \end{aligned}$$

Assuming that $\cos \theta \ne 0$, the pitch rotation can be generated from (13) as

16 $$\begin{aligned} \theta = \arctan \left( \frac{\left( U_1 + \frac{k_t}{m}\dot{x}\right) \cos \psi + \left( U_2 + \frac{k_t}{m}\dot{y}\right) \sin \psi }{U_3+g+\frac{k_t}{m}\dot{z}} \right) . \end{aligned}$$

Squaring both sides of (12) yields

17 $$\begin{aligned} \left( \frac{u}{m} z_e\right) ^{\tiny \textsf {T}}\left( \frac{u}{m} z_e\right) = \left( J_1^{-1}(\eta _2) \left( U + g z_e + \frac{k_t}{m} \dot{\eta }_1\right) \right) ^{\tiny \textsf {T}}\left( J_1^{-1}(\eta _2) \left( U + g z_e + \frac{k_t}{m} \dot{\eta }_1\right) \right) . \end{aligned}$$

As a result

18 $$\begin{aligned} \frac{u}{m} = \left( \left( U_1 + \frac{k_t}{m}\dot{x}\right) ^2 + \left( U_2 + \frac{k_t}{m}\dot{y}\right) ^2 + \left( U_3 + g + \frac{k_t}{m}\dot{z}\right) ^2 \right) ^{\!1/2} . \end{aligned}$$

From (14) and (15), we have

19 $$\begin{aligned} \frac{u}{m} \sin (\phi ) = \left( U_1 + \frac{k_t}{m}\dot{x}\right) \sin (\psi ) - \left( U_2 + \frac{k_t}{m}\dot{y}\right) \cos (\psi ) , \end{aligned}$$

and by substituting (18)–(19), the roll rotation can be derived as

20 $$\begin{aligned} \phi = \arcsin \left( \frac{\left( U_1 + \frac{k_t}{m}\dot{x}\right) \sin \psi - \left( U_2 + \frac{k_t}{m}\dot{y}\right) \cos \psi }{ \sqrt{\left( U_1 + \frac{k_t}{m}\dot{x}\right) ^2 + \left( U_2 + \frac{k_t}{m}\dot{y}\right) ^2 + \left( U_3 + g + \frac{k_t}{m}\dot{z}\right) ^2} } \right) . \end{aligned}$$

From (15), the total thrust can be computed as follows

21 $$\begin{aligned} u&= m \left( \left( U_1 + \frac{k_t}{m}\dot{x}\right) (\cos \phi \sin \theta \cos \psi + \sin \phi \sin \psi ) + \left( U_2 + \frac{k_t}{m}\dot{y}\right) (\cos \phi \sin \theta \sin \psi - \sin \phi \cos \psi ) \right. \nonumber \\&\quad \left. + \left( U_3+g+\frac{k_t}{m}\dot{z}\right) \cos \phi \cos \theta \right) . \end{aligned}$$

The desired roll $\phi _d$ and pitch $\theta _d$ angles can be calculated from (20) and (16) is a similar way

22 $$\begin{aligned} \phi _d&= \arcsin \left( \frac{\left( U_1 + \frac{k_t}{m}\dot{x}_d\right) \sin \psi _d - \left( U_2 + \frac{k_t}{m}\dot{y}_d\right) \cos \psi _d}{ \sqrt{\left( U_1 + \frac{k_t}{m}\dot{x}_d\right) ^2 + \left( U_2 + \frac{k_t}{m}\dot{y}_d\right) ^2 + \left( U_3 + g + \frac{k_t}{m}\dot{z}_d\right) ^2} } \right) , \end{aligned}$$

23 $$\begin{aligned} \theta _d&=\arctan \left( \frac{\left( U_1 + \frac{k_t}{m}\dot{x}_d\right) \cos \psi _d + \left( U_2 + \frac{k_t}{m}\dot{y}_d\right) \sin \psi _d}{U_3+g+\frac{k_t}{m} \dot{z}_d} \right) . \end{aligned}$$

### Attitude control design

In this section, the main contribution of the paper is explained. The presence of uncertain parameters in the attitude dynamics will make the attitude control of UAV a challenging problem. If all parameters of the system are known for the feedback control design, the nonlinear terms could be cancelled by introducing a simple feedback linearization technique. However, in many practical situations, several parameters of the UAV such as $I_R$, $k_r$ and $\delta$ are often unknown. Consequently, a proper controller such as adaptive techniques is required to handle the uncertainties. CAS, relying on the certainty equivalence principle, has two major steps in the design procedure. First, a controller is designed for the system without any uncertain parameters. In this way, uncertain parameters are assumed to be readily available for the feedback control design. In the second step, the parameters estimated by the adaptive law are used as the values of the uncertain parameters.

Before presenting the main results, the desired trajectory is defined as $$\nu _{2_d} = \begin{bmatrix} p_d&q_d&r_d \end{bmatrix}^{\tiny \textsf {T}}\in \mathbb {R}^{3\times 1}$$. Therefore, the trajectory error can be calculated from $e = \nu _2 - \nu _{2_d}$. The reference model is introduced by

24 $$\begin{aligned} \dot{\hat{\nu }}_2 = -\alpha \hat{\nu }_2 + \alpha \nu _{2_d}, \end{aligned}$$

where $\alpha \in \mathbb {R}$ is a positive constant and $\hat{\nu }_2$ is the state of the reference model. This dynamics leads to the desired trajectory $\nu _{2_d}$. Hence, the tracking error dynamics (7) and (24) can be written as

25 $$\begin{aligned} \dot{\tilde{\nu }}_2 = \alpha (\hat{\nu }_2 - \nu _{2_d}) + f(\nu _2) + g_1(\nu _2)I_R + g_2(\nu _2)k_r + h \delta + I_{M}^{-1} \tau , \end{aligned}$$

where $$\tilde{\nu }_2 = \nu _2 - \hat{\nu }_2$$.

#### A classical adaptive scheme

In the first scheme, we present CAS to estimate the uncertain parameters in the attitude dynamics. Let $\hat{I}_R (t) \in \mathbb {R}$, $\hat{k}_r (t) \in \mathbb {R}$ and $\hat{\delta }(t) \in \mathbb {R}$ be the estimation of unknown parameters $I_R$, $k_r$ and $\delta$, respectively. The adaptation law is deemed to be successful if

26 $$\begin{aligned} \lim _{t\rightarrow \infty } \tilde{I}_R (t),\tilde{k}_r (t),\tilde{\delta }(t) = 0 , \end{aligned}$$

where $\tilde{I}_R (t) = \hat{I}_R (t)-I_R$, $\tilde{k}_r (t)=\hat{k}_r (t)-k_r$ and $\tilde{\delta }(t) = \hat{\delta }(t)-\delta$. CAS is presented in Theorem 1.

**Figure 2:**
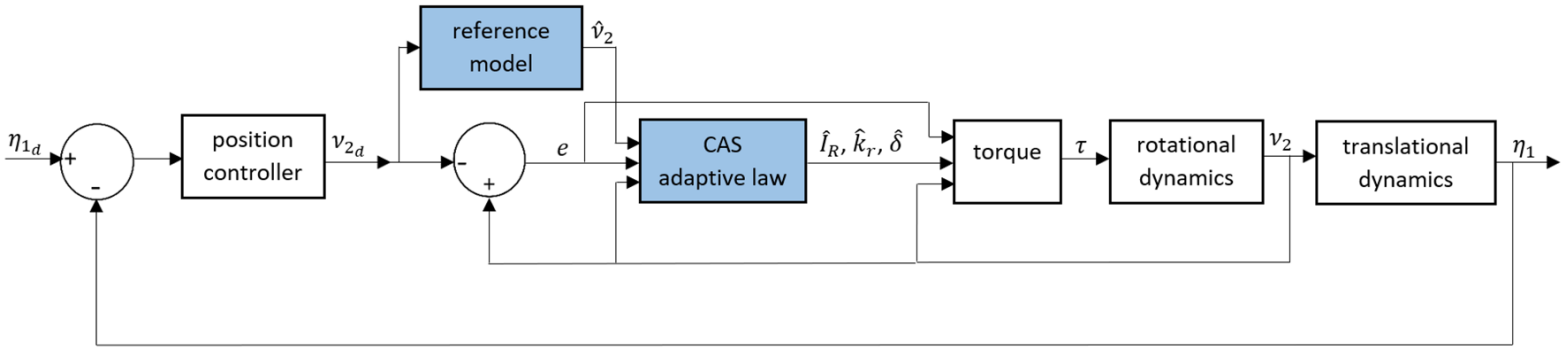
The control system design for a 6-DOF UAV using CAS.

**Figure 3:**
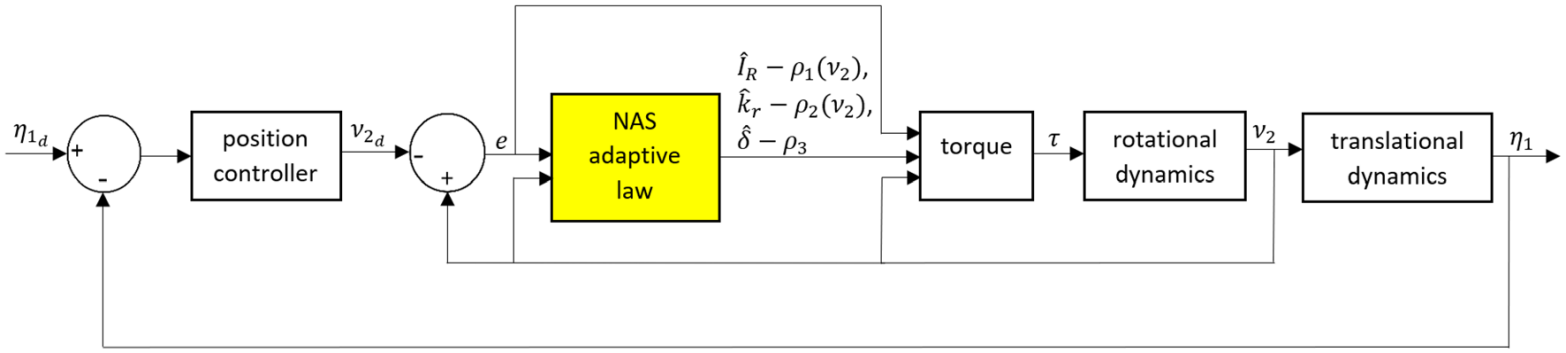
The control system design for a 6-DOF UAV using NAS.

##### **Theorem 1**

(CAS) Consider the attitude dynamics (7). The proposed control law is designed as

27 $$\begin{aligned} \tau = I_M \left( - \alpha e - f(\nu _2) - g_1(\nu _2) \hat{I}_R - g_2(\nu _2) \hat{k}_r - h \hat{\delta }\right) , \end{aligned}$$

where $\hat{I}_R$, $\hat{k}_r$, $\hat{\delta }$ are updated by the following adaptation laws

28 $$\begin{aligned} \dot{\hat{I}}_R&= \gamma _1 \tilde{\nu }_2^{\tiny \textsf {T}}g_1(\nu _2), \nonumber \\ \dot{\hat{k}}_r&= \gamma _2 \tilde{\nu }_2^{\tiny \textsf {T}}g_2(\nu _2), \nonumber \\ \dot{\hat{\delta }}&= \gamma _3 \tilde{\nu }_2^{\tiny \textsf {T}}h, \end{aligned}$$

for some $\alpha , \gamma _1, \gamma _2, \gamma _3 > 0$ and $\tilde{\nu }_2 = \nu _2 - \hat{\nu }_2$. Then the time-derivative of

29 $$\begin{aligned} V(\tilde{\nu }_2,\tilde{I}_R,\tilde{k}_r,\tilde{\delta }) = \frac{1}{2} \tilde{\nu }_2^{\tiny \textsf {T}}\tilde{\nu }_2 + \frac{1}{2\gamma _1} \tilde{I}_R^2 + \frac{1}{2\gamma _2} \tilde{k}_r^2 + \frac{1}{2\gamma _3} \tilde{\delta }^2 , \end{aligned}$$

along the trajectories of the closed-loop system (7) + (27) + (28) is

30 $$\begin{aligned} \dot{V}(\tilde{\nu }_2,\tilde{I}_R,\tilde{k}_r,\tilde{\delta }) = -\alpha \tilde{\nu }_2^{\tiny \textsf {T}}\tilde{\nu }_2 . \end{aligned}$$

##### *Proof*

The error dynamic of the closed-loop system (7) + (27) + (28) can be written as

31 $$\begin{aligned} \dot{\tilde{\nu }}_2 = -\alpha \tilde{\nu }_2 - g_1(\nu _2)(\hat{I}_R-I_R) - g_2(\nu _2)(\hat{k}_r-k_r) - h(\nu _2)(\hat{\delta }-\delta ) . \end{aligned}$$

Direct calculation shows that the time-derivative of $V(\tilde{\nu }_2,\tilde{I}_R,\tilde{k}_r,\tilde{\delta })$ is

32 $$\begin{aligned} \dot{V}(\tilde{\nu }_2,\tilde{I}_R,\tilde{k}_r,\tilde{\delta })&= \tilde{\nu }_2^{\tiny \textsf {T}}\dot{\tilde{\nu }}_2 + \frac{1}{\gamma _1} \dot{\hat{I}}_R (\hat{I}_R-I_R) + \frac{1}{\gamma _2} \dot{\hat{k}}_r (\hat{k}_r-k_r) + \frac{1}{\gamma _3} \dot{\hat{\delta }} (\hat{\delta }-\delta ) \nonumber \\&= \tilde{\nu }_2^{\tiny \textsf {T}}\left( -\alpha \tilde{\nu }_2 - g_1(\nu _2)(\hat{I}_R-I_R) - g_2(\nu _2)(\hat{k}_r-k_r) - h(\nu _2)(\hat{\delta }-\delta ) \right) + \tilde{\nu }_2^{\tiny \textsf {T}}g_1(\nu _2) (\hat{I}_R-I_R) \nonumber \\&\quad + \tilde{\nu }_2^{\tiny \textsf {T}}g_2(\nu _2)(\hat{k}_r-k_r) + \tilde{\nu }_2^{\tiny \textsf {T}}h (\hat{\delta }-\delta ) \nonumber \\&= -\alpha \tilde{\nu }_2^{\tiny \textsf {T}}\tilde{\nu }_2 - \tilde{\nu }_2^{\tiny \textsf {T}}g_1(\nu _2)(\hat{I}_R-I_R) - \tilde{\nu }_2^{\tiny \textsf {T}}g_2(\nu _2)(\hat{k}_r-k_r) - \tilde{\nu }_2^{\tiny \textsf {T}}h(\nu _2)(\hat{\delta }-\delta ) + \tilde{\nu }_2^{\tiny \textsf {T}}g_1(\nu _2) (\hat{I}_R-I_R) \nonumber \\&\quad + \tilde{\nu }_2^{\tiny \textsf {T}}g_2(\nu _2) (\hat{k}_r-k_r) + \tilde{\nu }_2^{\tiny \textsf {T}}h (\hat{\delta }-\delta ) \nonumber \\&= -\alpha \tilde{\nu }_2^{\tiny \textsf {T}}\tilde{\nu }_2 . \end{aligned}$$

From (28) and (31), we can see that $\tilde{\nu }_2(t)$, $\tilde{I}_R$, $\tilde{k}_r$, and $\tilde{\delta }$ are bounded. To show the tracking error $\tilde{\nu }_2$ is driven asymptotically to zero, we calculate the second time-derivative of Lyapunov function $\dot{V}(\tilde{\nu }_2,\tilde{I}_R,\tilde{k}_r,\tilde{\delta })$ as

33 $$\begin{aligned} \ddot{V}(\tilde{\nu }_2,\tilde{I}_R,\tilde{k}_r,\tilde{\delta }) = -2 \alpha \tilde{\nu }_2^{\tiny \textsf {T}}\dot{\tilde{\nu }}_2. \end{aligned}$$

From (31) it can be seen that $\tilde{\nu }_2$ is uniformly bounded, and hence $\ddot{V}(\tilde{\nu }_2,\tilde{I}_R,\tilde{k}_r,\tilde{\delta })$ is bounded. This implies that $\dot{V}(\tilde{\nu }_2,\tilde{I}_R,\tilde{k}_r,\tilde{\delta })$ is uniformly continuous. By using Barbalat’s Lemma, we have $\lim _{t\rightarrow \infty } \tilde{\nu }_2(t) = 0$, and this implies that $\lim _{t\rightarrow \infty } e(t) = 0$. This completes the proof of the theorem. $\square$

It can be observed from (32) that the decay rate of the Lyapunov function depends on the model reference tracking error $\tilde{\nu }_2(t)$ and we can guarantee the stability of the close-loop system even without having $\tilde{\nu }_2(t)$ equals zero. On the other hand, looking at the adaptation law (28) along with the Lyapunov function (29) clarifies that since we are minimising all errors together for a non-zero $\tilde{\nu }_2(t)$, the adaptive rule updates itself constantly even the estimated parameters reach the actual value of the unknown parameter. Nonetheless, when $\tilde{\nu }_2(t)$ converges to zero, we see from (28) that the estimated parameters converge to a constant value and there is no change in the Lyapunov function according to (30) and this may lead to a non-zero Lyapunov function in (29). Consequently, the convergence of the parameter estimation error to zero cannot always be guaranteed using the controller proposed in Theorem 1. Moreover, due to existent of $\hat{\nu }_2$ for the feedback control design, the controller is not economical for practical implementation. The schematic block diagram of the UAV control system designed according to Theorem 1 is presented in Fig. 2. Although the extra state in (24) can be removed from the control system structure, using CAS^14^, the controller in^14^ suffers from the numerical stability due to the presence of the tracking error derivatives.

#### A new adaptive scheme

To address the issues mentioned in the previous section, NAS is proposed in this section to handle the uncertain parameters in the attitude dynamic of UAV. NAS is proposed in Theorem 2 below.

##### **Theorem 2**

(NAS) Consider the attitude dynamic in (7). The control law is designed to be

34 $$\begin{aligned} \tau = I_M \left( - \alpha e - f(\nu _2) - g_1(\nu _2) (\hat{I}_R - \rho _1(\nu _2)) - g_2(\nu _2) (\hat{k}_r - \rho _2(\nu _2)) - h (\hat{\delta }- \rho _3(\nu _2)) \right) , \end{aligned}$$

where $\hat{I}_R$, $\hat{k}_r$, $\hat{\delta }$ are updated by

35 $$\begin{aligned} \dot{\hat{I}}_R&= \alpha \lambda _1 g_{1}^{\tiny \textsf {T}}(\nu _2)e, \nonumber \\ \dot{\hat{k}}_r&= \alpha \lambda _2 g_{2}^{\tiny \textsf {T}}(\nu _2)e, \nonumber \\ \dot{\hat{\delta }}&= \alpha \lambda _3 h^{\tiny \textsf {T}}e, \end{aligned}$$

for some positive constants $\alpha , \lambda _1, \lambda _2, \lambda _3$; and $\rho _1(\nu _2) \in \mathbb {R}$, $\rho _2(\nu _2) \in \mathbb {R}$ and $\rho _3 \in \mathbb {R}$ are any continuous differentiable functions satisfying

36 $$\begin{aligned} \frac{\partial \rho _1(\nu _2) }{\partial \nu _2}&= -\lambda _1 g_{1}^{\tiny \textsf {T}}(\nu _2), \nonumber \\ \frac{\partial \rho _2(\nu _2) }{\partial \nu _2}&= -\lambda _2 g_{2}^{\tiny \textsf {T}}(\nu _2), \nonumber \\ \frac{\partial \rho _3(t) }{\partial t}&= -\lambda _3 h^{\tiny \textsf {T}}, \end{aligned}$$

then the time-derivative of

37 $$\begin{aligned} V(\tilde{\nu }_2,z_1,z_2,z_3) = \frac{1}{2} \tilde{\nu }_2^{\tiny \textsf {T}}\tilde{\nu }_2 + \frac{\sigma }{4(1-k)} \left( \frac{z_1^2}{2\lambda _1} + \frac{z_2^2}{2\lambda _2} + \frac{z_3^2}{2\lambda _3} \right) , \end{aligned}$$

with

38 $$\begin{aligned} \frac{\tilde{\nu }_2^{\tiny \textsf {T}}\tilde{\nu }_2}{\alpha \tilde{\nu }_2^{\tiny \textsf {T}}\tilde{\nu }_2} \le \sigma < \infty , \end{aligned}$$

where $\alpha$ and $\sigma$ are some positive constants, $0<k<1$, $z_1=\rho _1(\nu _2)-\hat{I}_R+I_R$, $z_2=\rho _2(\nu _2)-\hat{k}_r+k_r$ and $z_3=\rho _3-\hat{\delta }+\delta$. Then the derivative of the Lyapunov function (37) along with the trajectories of the closed-loop system (7) + (34) + (35) satisfies

39 $$\begin{aligned} \dot{V}(\tilde{\nu }_2,z_1,z_2,z_3) \le -k\alpha \tilde{\nu }_2^{\tiny \textsf {T}}\tilde{\nu }_2. \end{aligned}$$

##### *Proof*

The error dynamic of the closed-loop system (7)+(34)+(35) can be written as

40 $$\begin{aligned} \dot{\tilde{\nu }}_2 = -\alpha \tilde{\nu }_2 + g_1(\nu _2)z_1 + g_2(\nu _2)z_2 + h z_3. \end{aligned}$$

In this case, the Lyapunov function can be rewritten as

41 $$\begin{aligned} V(\tilde{\nu }_2,z_1,z_2,z_3) = V_{\tilde{\nu }_2}(\tilde{\nu }_2) + \frac{\sigma }{4(1-k)} \left( V_{z_1}(z_1) + V_{z_2}(z_2) + V_{z_3}(z_3) \right) , \end{aligned}$$

where

$$\begin{aligned} V_{z_1}(z_1) = \frac{1}{2 \lambda _1} z_1^2, \;V_{z_2}(z_2) = \frac{1}{2 \lambda _2} z_2^2, \;V_{z_3}(z_3) = \frac{1}{2 \lambda _3} z_3^2. \end{aligned}$$

Direct calculation shows that the time-derivative of $V_{\tilde{\nu }_2}(\tilde{\nu }_2)$ along the system dynamic (40) is

$$\begin{aligned} \dot{V}_{\tilde{\nu }_2}(\tilde{\nu }_2)&= \tilde{\nu }_2^{\tiny \textsf {T}}\dot{\tilde{\nu }}_2 \\&= \tilde{\nu }_2^{\tiny \textsf {T}}\left( -\alpha \tilde{\nu }_2 + g_1(\nu _2)z_1 + g_2(\nu _2)z_2 + h z_3\right) \\&= -\alpha \tilde{\nu }_2^{\tiny \textsf {T}}\tilde{\nu }_2 + \tilde{\nu }_2^{\tiny \textsf {T}}\left( g_1(\nu _2)z_1 + g_2(\nu _2)z_2 + h z_3\right) . \end{aligned}$$

By picking $a=\frac{1-k}{\sigma }$ and for any $0<k<1$, then we have

$$\begin{aligned} a \tilde{\nu }_2^{\tiny \textsf {T}}\tilde{\nu }_2 \le \alpha (1-k) \tilde{\nu }_2^{\tiny \textsf {T}}\tilde{\nu }_2 , \end{aligned}$$

as a result

$$\begin{aligned} \dot{V}_{\tilde{\nu }_2}(\tilde{\nu }_2)&\le -\alpha \tilde{\nu }_2^{\tiny \textsf {T}}\tilde{\nu }_2 + \alpha (1-k) \tilde{\nu }_2^{\tiny \textsf {T}}\tilde{\nu }_2 + \frac{1}{4a} \left( g_1(\nu _2)z_1 + g_2(\nu _2)z_2 + h z_3\right) ^2\\&\le -k \alpha \tilde{\nu }_2^{\tiny \textsf {T}}\tilde{\nu }_2 + \frac{1}{4a} \left( g_1(\nu _2)z_1 + g_2(\nu _2)z_2 + h z_3\right) ^2 . \end{aligned}$$

The time-derivative of $V_{z_1}(z_1)$, $V_{z_2}(z_2)$ and $V_{z_3}(z_3)$ can be computed as follows

$$\begin{aligned} \dot{V}_{z_1}(z_1)&= \frac{1}{\lambda _1} z_1 \dot{z}_1 \\&= \frac{1}{\lambda _1} z_1 \left( \frac{\partial \rho _1(\nu _2)}{\partial \nu _2} \dot{\nu }_2 - \dot{\hat{I}}_R \right) \\&= \frac{1}{\lambda _1} z_1 \left( \frac{\partial \rho _1(\nu _2)}{\partial \nu _2} ( f(\nu _2) + g_1(\nu _2)I_R + g_2(\nu _2)k_r + h \delta + I_{M}^{-1} \tau ) - \dot{\hat{I}}_R \right) \\&= \frac{1}{\lambda _1} z_1 \left( -\lambda _1 g_{1}^{\tiny \textsf {T}}(\nu _2) ( -\alpha e + g_1(\nu _2)z_1 + g_2(\nu _2)z_2 + h z_3 ) - \alpha \lambda _1 g_{1}^{\tiny \textsf {T}}(\nu _2)e \right) \\&= -g_{1}^{\tiny \textsf {T}}(\nu _2)z_1 ( g_1(\nu _2)z_1 + g_2(\nu _2)z_2 + h z_3 ) \\&= -g_1(\nu _2)^{\tiny \textsf {T}}g_1(\nu _2) z_1^2 - g_{1}^{\tiny \textsf {T}}(\nu _2)g_2(\nu _2)z_1 z_2 - g_{1}^{\tiny \textsf {T}}(\nu _2)hz_1 z_3 ,\\ \dot{V}_{z_2}(z_2)&= \frac{1}{\lambda _2} z_2 \dot{z}_2 \\&= \frac{1}{\lambda _2} z_2 \left( \frac{\partial \rho _2(\nu _2)}{\partial \nu _2} \dot{\nu }_2 - \dot{\hat{k}}_r \right) \\&= \frac{1}{\lambda _2} z_2 \left( \frac{\partial \rho _2(\nu _2)}{\partial \nu _2} \left( f(\nu _2) + g_1(\nu _2)I_R + g_2(\nu _2)k_r + h \delta + I_{M}^{-1} \tau \right) - \dot{\hat{k}}_r \right) \\&= \frac{1}{\lambda _2} z_2 \left( -\lambda _2 g_{2}^{\tiny \textsf {T}}(\nu _2) \left( -\alpha e + g_1(\nu _2)z_1 + g_2(\nu _2)z_2 + h z_3 \right) - \alpha \lambda _2 g_{2}^{\tiny \textsf {T}}(\nu _2)e \right) \\&= -g_{2}^{\tiny \textsf {T}}(\nu _2)z_2 ( g_1(\nu _2)z_1 + g_2(\nu _2)z_2 + h z_3 ) \\&= -g_2(\nu _2)^{\tiny \textsf {T}}g_2(\nu _2) z_2^2 - g_{1}^{\tiny \textsf {T}}(\nu _2)g_2(\nu _2)z_1 z_2 - g_{2}^{\tiny \textsf {T}}(\nu _2)hz_2 z_3 ,\\ \dot{V}_{z_3}(z_3)&= \frac{1}{\lambda _3} z_3 \dot{z}_3 \\&= \frac{1}{\lambda _3} z_3 \left( \frac{\partial \rho _3(\nu _2)}{\partial \nu _2} \dot{\nu }_2 - \dot{\hat{\delta }} \right) \\&= \frac{1}{\lambda _3} z_3 \left( \frac{\partial \rho _3(\nu _2)}{\partial \nu _2} \left( f(\nu _2) + g_1(\nu _2)I_R + g_2(\nu _2)k_r + h \delta + I_{M}^{-1} \tau \right) - \dot{\hat{\delta }} \right) \\&= \frac{1}{\lambda _3} z_3 \left( -\lambda _3 h^{\tiny \textsf {T}}( -\alpha e + g_1(\nu _2)z_1 + g_2(\nu _2)z_2 + h z_3 ) - \alpha \lambda _3 h^{\tiny \textsf {T}}e \right) \\&= -h^{\tiny \textsf {T}}z_3 ( g_1(\nu _2)z_1 + g_2(\nu _2)z_2 + h z_3 ) \\&= -h^{\tiny \textsf {T}}h z_3^2 - g_{1}^{\tiny \textsf {T}}(\nu _2)hz_1 z_3 - g_{2}^{\tiny \textsf {T}}(\nu _2)hz_2 z_3 , \end{aligned}$$

As a result, the time-derivative of $V(\tilde{\nu }_2,z_1,z_2,z_3)$ is

$$\begin{aligned} \dot{V}(\tilde{\nu }_2,z_1,z_2,z_3)&\le -k \alpha \tilde{\nu }_2^{\tiny \textsf {T}}\tilde{\nu }_2 + \frac{1}{4a} \left( g_1(\nu _2)z_1 + g_2(\nu _2)z_2 + h z_3 \right) ^2 + \frac{1}{4a} \left( -g_1(\nu _2)^{\tiny \textsf {T}}g_1(\nu _2) z_1^2 - g_2(\nu _2)^{\tiny \textsf {T}}g_2(\nu _2) z_2^2 \right. \\&\quad \left. - h^{\tiny \textsf {T}}h z_3^2 - 2g_{1}^{\tiny \textsf {T}}(\nu _2)g_2(\nu _2)z_1 z_2 - 2g_{1}^{\tiny \textsf {T}}(\nu _2)hz_1 z_3 - 2g_{2}^{\tiny \textsf {T}}(\nu _2)hz_2 z_3 \right) \\&\le -k \alpha \tilde{\nu }_2^{\tiny \textsf {T}}\tilde{\nu }_2 + \frac{1}{4a} \left( g_1(\nu _2)z_1 + g_2(\nu _2)z_2 + h z_3 \right) ^2 - \frac{1}{4a} \left( g_1(\nu _2)z_1 + g_2(\nu _2)z_2 + h z_3\right) ^2 \\&\le -k \alpha \tilde{\nu }_2^{\tiny \textsf {T}}\tilde{\nu }_2 . \end{aligned}$$

Thus, the proof is completed. $\square$

##### *Remark 1*

Compared to CAS, the estimated parameters $\hat{I}_R$, $\hat{k}_r$ and $\hat{\delta }$ are not converging directly to the real value of $I_R$, $k_r$ and $\delta$, but they converge to $I_R + \rho _1(\nu _2)$, $k_r + \rho _2(\nu _2)$ and $\delta + \rho _3$, respectively, for a designed functions $\rho _1(\nu _2)$, $\rho _2(\nu _2)$ and $\rho _3$.

##### *Remark 2*

The functions of $\rho _1(\nu _2) \in \mathbb {R}$, $\rho _2(\nu _2) \in \mathbb {R}$ and $\rho _3 \in \mathbb {R}$ are any continuous differentiable functions computed from (36). For example, $\rho _1(\nu _2)$, $\rho _2(\nu _2)$ and $\rho _3$ can be selected as follows

42 $$\begin{aligned} \rho _1(\nu _2)&= -\lambda _1 g_{1}^{\tiny \textsf {T}}(\nu _2) \nu _2 + c_1, \nonumber \\ \rho _2(\nu _2)&= -\lambda _2 g_{2}^{\tiny \textsf {T}}(\nu _2) \nu _2 + c_2, \nonumber \\ \rho _3(t)&= -\lambda _3 h^{\tiny \textsf {T}}\begin{bmatrix} t&t&t \end{bmatrix}^{\tiny \textsf {T}}+ c_3 , \end{aligned}$$

where $c_1$, $c_2$ and $c_3$ are some constants.

##### *Remark 3*

From Theorem 2, it can be observed that $z_1$, $z_2$, and $z_3$ converge to an appropriate manifold as $\dot{V}_{z_1}(z_1)$, $\dot{V}_{z_2}(z_2)$, and $\dot{V}_{z_3}(z_3)$ are always negative for any $z_1$, $z_2$, and $z_3 \ne 0$. In this way, the open problem in the CAS is solved. The schematic block diagram of NAS, proposed in Theorem 2 is illustrated in Fig. 3. By comparing Figs. 2 and 3, we can see that the control structure in NAS is simpler than CAS. This is due to the fact that the reference model is required in CAS to avoid $\dot{\nu }_{2_d}$ for the feedback control design and generate $\tilde{\nu }_2$ instead in the adaptation law in (28). The presence of $\dot{\nu }_{2_d}$ in the control input may cause some numerical issues in both simulation and practical results. Nonetheless, the NAS is designed in a way that $\tilde{\nu }_2$ is only required for the stability analysis and it doesn’t appear in the adaptation law in (35).

## Simulation and experimental results

In this section, the performance of our designed control system is evaluated numerically and demonstrated by practical implementation on a real drone. A virtual PD controller is implemented numerically as the translational controller. One way to design the gains of the PD controller is suggested in “Translational control design” section. The tuned values for these gains are $K_P=K_D=\text {diag}(1)$. The control gains for the rotational motion are designed according to the Theorems 1 and 2. The gains of (27) and (28) are tuned to be

$$\begin{aligned} \alpha = 1000, \;\gamma _1 = 1000, \; \gamma _2 = 0.0001, \;\gamma _3 = 20 . \end{aligned}$$

The gains of the controller in (34) and (35) are

$$\begin{aligned} \alpha = 1000, \;\lambda _1 = 100, \; \lambda _2 = 0.00001, \;\lambda _3 = 2. \end{aligned}$$

These gains $\alpha$, $\gamma _1$, $\gamma _2$ and $\gamma _3$ are usually tuned on a case-by-case basis and looking at the system response. Generally speaking, the higher the gains, the better the steady-state tracking results will be. However, we should always consider a trade-off to avoid control actuator saturation due to high gain or amplify measurement noises in the system outputs.

**Figure 4:**
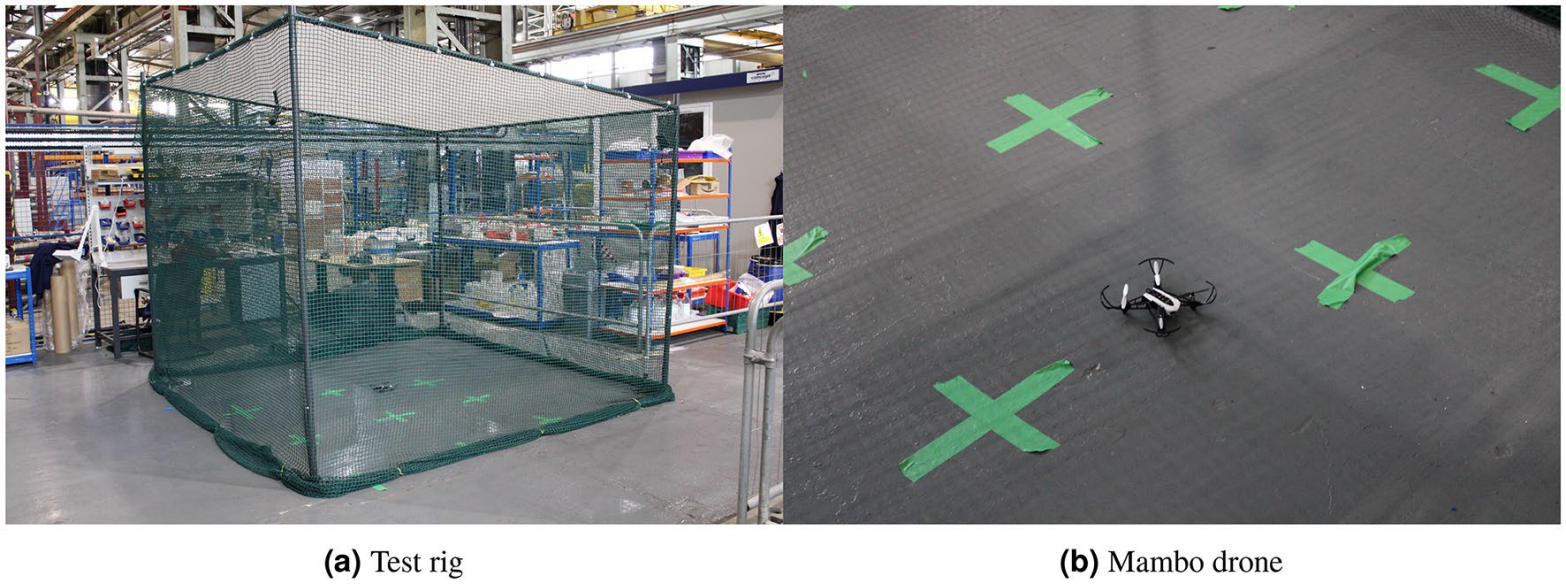
The hardware platform used for experimental verification of the proposed adaptive scheme.

The external disturbance $$h=\begin{bmatrix} \sin (t)&0.8\sin (t)&0.6\sin (t) \end{bmatrix}^{\tiny \textsf {T}}$$ with an unknown constant $\delta =0.2$ is added to the system dynamics. The parameters of the UAV used for both numerical simulation and practical implementation of the controller are listed in Table 1. The hardware platform used in this experiment is the parrot mambo minidrone as illustrated in Fig. 4.

**Table 1:** The parameters of the mini-drone.

Parameter name	Notation	Value
Mass	*m*	0.063 kg
Gravity acceleration	*g*	9.8 m/s$^2$
Translational drag coefficient	$k_t$	0
Rotational drag coefficient	$k_r$	0.02
Arm length	*l*	0.0624 m
Drag factor	*d*	0.0024 kg m$^2$g
Propeller inertia	$I_R$	1.0209e−2 kg m$^2$
Inertia of *x*-axis	$I_x$	0.0686e−3 kg $m^2$
Inertia of *y*-axis	$I_y$	0.092e−3 kg m$^2$
Inertia of *z*-axis	$I_z$	0.1366e−3 kg m$^2$

**Figure 5:**
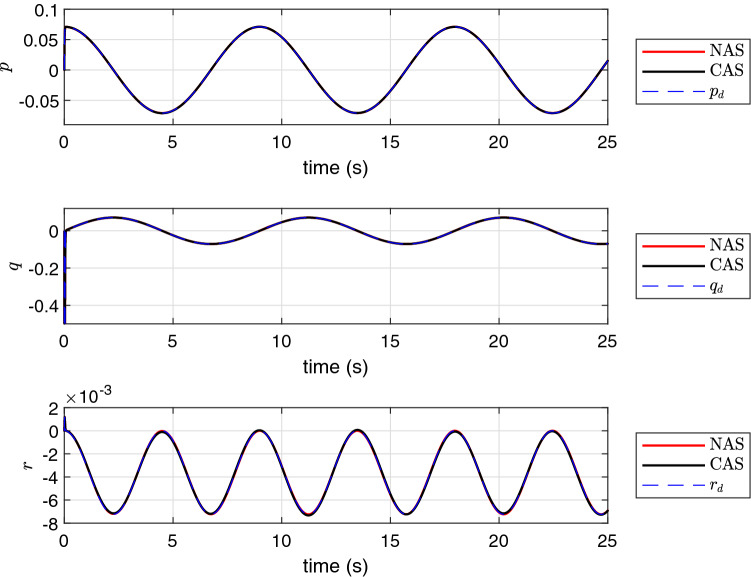
The profile of *p*, *q* and *r*.

**Figure 6:**
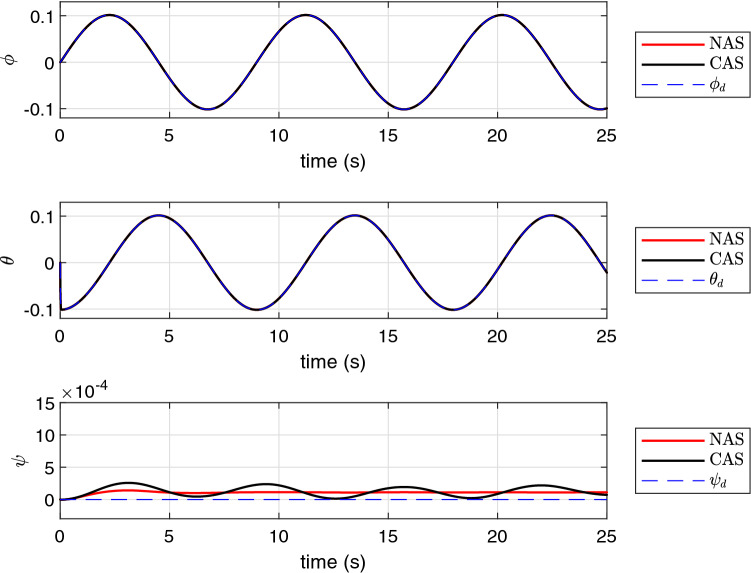
The profile of $\phi$, $\theta$ and $\psi$.

**Figure 7:**
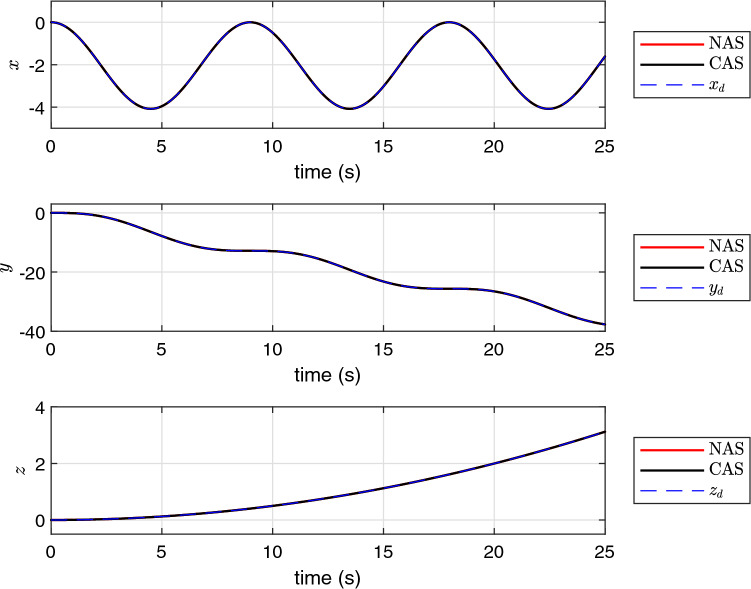
The profile of *x*, *y* and *z*.

**Figure 8:**
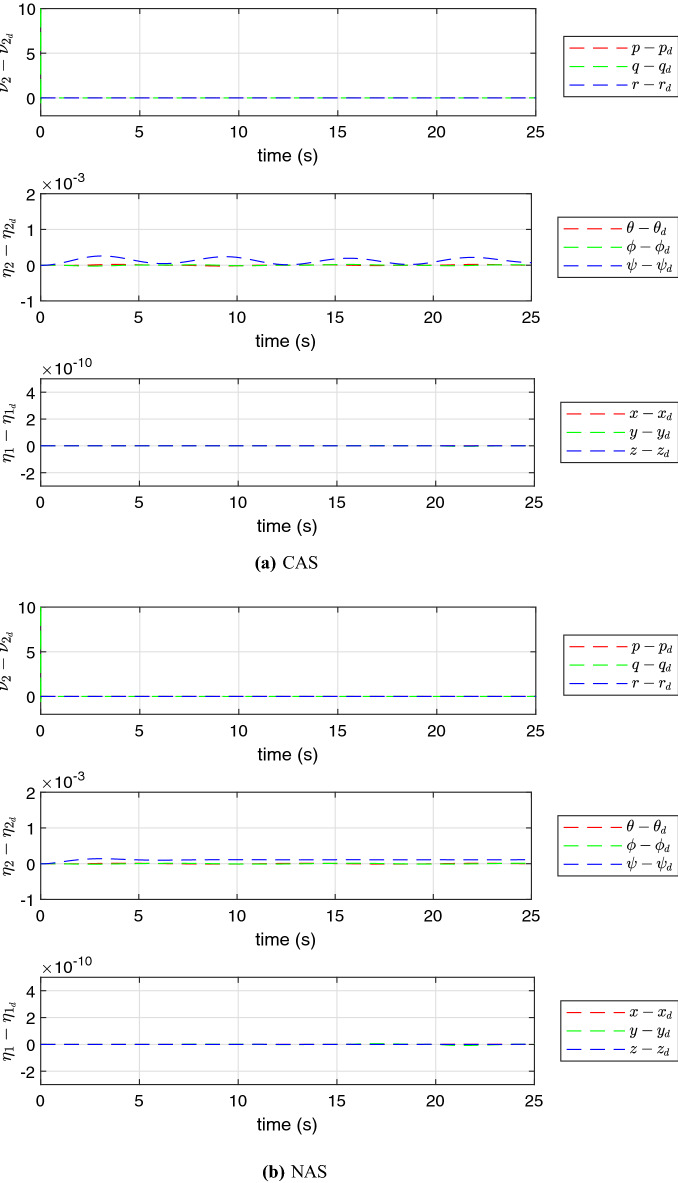
The profile of the trajectory tracking errors.

**Figure 9:**
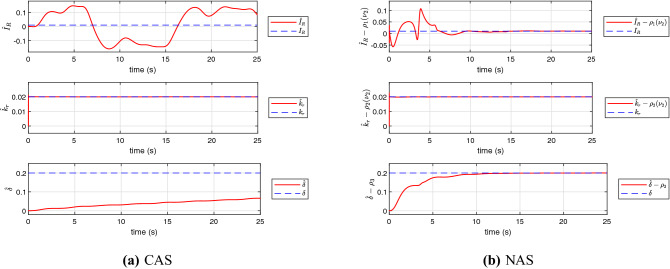
The state profile of the adaptive controller to handle uncertain parameters.

The simulation results using the proposed schemes are illustrated in Figs. 5, 6 and 7. The trajectory tracking errors for these results are plotted in Fig. 8. From the figures, we can see that both translational and rotational states of the quadrotor can follow the desired trajectories. This verifies the performance of the controller developed in “Proposed control design” section. The convergence of the parameter estimation errors is illustrated in Fig. 9 . We can see from this figure that NAS is capable in driving the parameter estimation errors to zero. However, CAS is not successful in converging the parameter estimation errors to their actual values. This is an example of the situation where there is no guarantee that the estimated parameters converge to the actual value of unknown parameters or perhaps the algorithm needs more time to converge to zero using CAS. Basically, this is caused by the inherent drawback of CAS, in which the parameter estimation error relies on the model tracking error.

In order to evaluate the performance of the proposed algorithm compared to the existing methods, various robust and adaptive control methods in which the system uncertainties are dominated have been reviewed in “Related works and main contributions” section. In these approaches only the tracking controller is designed to maintain the effect of the uncertainties on the system performance in a certain bound, however, in all these scenarios the unknown parameters of the UAV are not estimated. The classical adaptive method, i.e. Theorem 1, is developed using the certainty equivalence principle, and hence the boundedness assumption on the uncertainties is removed. The adaptive controller is not able to guarantee the estimated parameters converge to the actual value of the unknown parameters. This is an inherent drawback of the standard/classical adaptive method. However, in NAS, a new adaptive controller by adding an extra continuous derivative function is proposed to assist the algorithm to find the right direction by estimating the true value of parameters. NAS guarantees handling the parametric uncertainties with an appropriate design manifold. In the literature this problem is dealt with by linearizing the nonlinear term with unknown parameter. Therefore, the difficulty in estimating the unknown parameters of the system in a nonlinear dynamics is avoided. Another method proposed in the literature is by using intelligent computation such as neural networks and genetic algorithms. The tracking control and parameter estimation are achieved but with some level of residual error. A qualitative comparison of these different approaches is summarised in Table 2.

**Table 2:** Comparison of several existing methods and the proposed schemes.

Method	Trajectory tracking error	Parameter estimation error
Intelligent computation^24–26^	Achievable with residual error	Achievable with residual error
SMC^11,12^	Asymptotically achievable	Not achievable
Adaptive SMC^10^	Asymptotically achievable	Not achievable
model predictive control(MPC)^33^	Asymptotically achievable	Not achievable
CAS	Asymptotically achievable	No guarantee to be achievable
NAS	Asymptotically achievable	Asymptotically achievable

**Figure 10:**
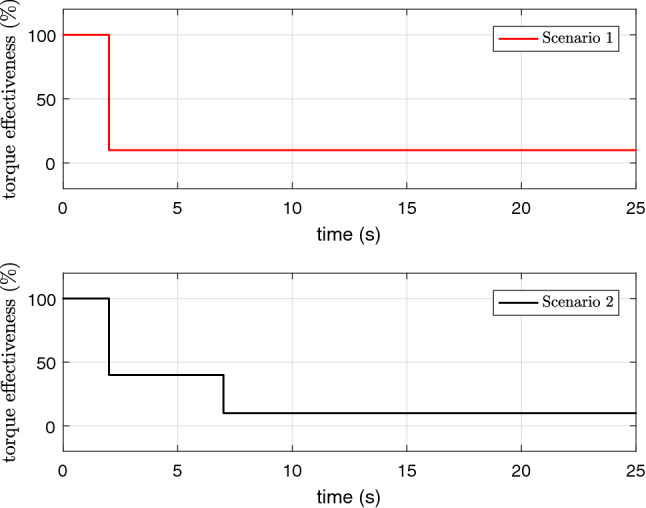
The profile of control effectiveness for two faults scenarios.

**Figure 11:**
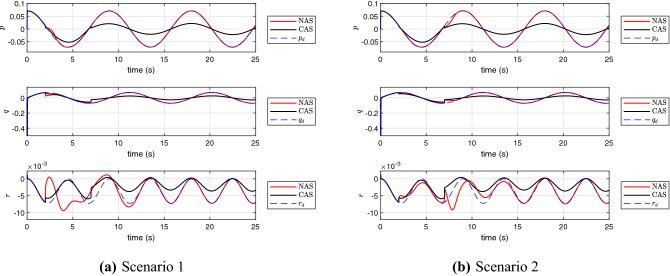
The profile of *p*, *q* and *r* in the presence of abrupt faults.

For further assessment of the proposed scheme in front of possible faults in the UAV, the algorithms are numerically evaluated for a scenario in which the UAV actuators experience an abrupt failure. The actuator faults can be represented by a constant parameter, named control effectiveness level of the actuator as explained in^34^. By changing this value between zero and one, it is possible to model different levels of actuator faults. Although the aim of this investigation is not to develop a fault tolerant control system with controller reconfiguration ability, here we evaluate the robustness of the controller for two scenarios for the actuator fault. These scenarios are illustrated in Fig. 10. In the first scenario, it is assumed that the UAV is operating with the nominal performance until 2*s* and all of the sudden four propellers are working with $10\%$ of the full torque value $\tau$. The parameters of the controllers are tuned to achieve the best nominal performance as shown in Fig. 5 and no changes on the control parameters is applied when an abrupt fault happens. The numerical results in Fig. 11a confirm that the angular rates in NAS is still capable to track the desired trajectories very closely, while CAS fails to maintain a reasonably well tracking performance for the UAV. In the second scenario, we assume multiple abrupt fault happens on $\tau$ and the control effectiveness level of this actuator is reduced. As before, the parameters of the controllers are tuned to achieve the best nominal performance according to Fig. 5 and no changes on the control parameters is applied when the abrupt faults happen. The numerical results in Fig. 11b show that the angular rates in NAS is still capable to track the desired trajectories very closely in this degradation scenario, while CAS fails to keep a good tracking performance for the UAV after the second level. For both scenarios the fitness of the tracking error in the steady state behavior of the rotational dynamic is calculated from $t=15s$ to $t=25s$ and the results are listed in Tables 3, 4, and 5. The fitness is calculated using the following formula

43 $$\begin{aligned} \text {fitness of } \nu _{2_i} (\%) = 100 \left( 1 - \frac{\Vert \nu _{{2_d}_i} - \nu _{2_i} \Vert }{\Vert \nu _{2_i} \Vert } \right) , \end{aligned}$$

where $\nu _{2_i}$ is the state of rotational dynamics and $\nu _{{2_d}_i}$ is the desired trajectory of $\nu _{2_i}$.

The simulation and fitness results, confirm that there are some major differences between NAS and CAS as listed below

### *Remark 4*

As can be seen from Fig. 9, the new scheme (NAS), is able to guarantee the convergence of unknown estimated parameters in the closed loop. There are many approaches proposed to estimate the unknown parameters, but the results for the systems with nonlinear dynamics are still limited in the literature as we stated in “Related works and main contributions” section (Related Works and Main Contributions). As a result, in NAS, we can generate an appropriate control input for the system by converging the adaptive estimations to the true values of the parameters while this is not the case for the CAS algorithm.

### *Remark 5*

As can be inferred from Tables 4 and 5 and Fig. 11 in the new revision, the NAS shows much more robustness against abrupt actuator faults thanks to the presence of the additional continuous function.

In order to validate the performance of the controller experimentally, a Parrot Mambo minidrone was used. The Mambo minidrone platform provides an ideal solution for rapid prototyping of quadrotor control systems. It uses a combination of ultrasonic, air pressure, optical flow, gyroscope, and accelerometer sensors in order to estimate its position and attitude. The states of the Mambo minidrone are estimated using a Kalman filter for sensor fusion. MATLAB and Simulink were used to develop the proposed control system using the Parrot Minidrones Support package. The support package also provides a simulation platform for safe testing of the control systems before deployment to the physical quadrotor. Once the controller was developed and tuned within MATLAB and Simulink, Simulink coder is used to convert the control system into C code, before uploading it wirelessly to the Mambo minidrone via Bluetooth connection. The control loop operates with a sample time of $T= 0.005s$. Data is then recorded on the Mambo minidrone for the duration of the flight. After the flight is completed, data from the flight can be downloaded to MATLAB via Bluetooth. Figure 4 shows the Mambo minidrone in the testing environment. This was a $3m \times 3m \times 2.4m$ gazebo with netting secured around it for safe testing of the Mambo minidrone. In addition to this, further software limitations were placed on the parrot minidrone to ensure safe operation. Firstly, saturation blocks were introduced on motor command signals to ensure that the signals remained within the maximum region for motor control. A crash detection feature was also present within the code that would cause the motors to shut off if the absolute value of *x* or *y* position of the minidrone was larger than a selected value. Finally, a crash would also be detected if the rate of change of the quadrotor positions was also above a selected value.

**Figure 12:**
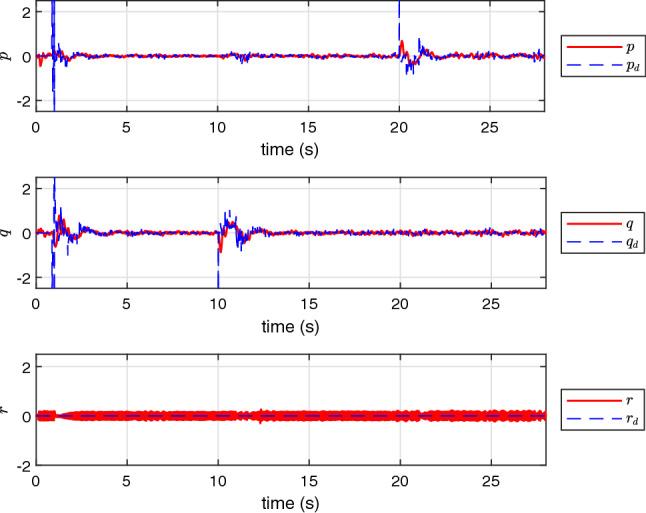
The profile of *p*, *q* and *r* in the experimental test using NAS.

**Figure 13:**
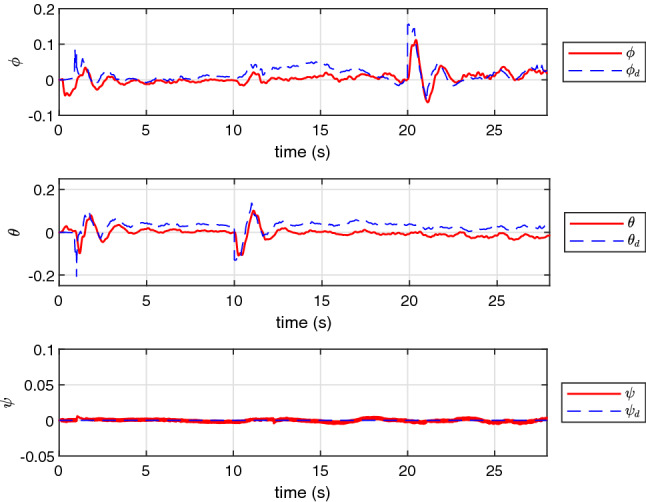
The profile of $\phi$, $\theta$ and $\psi$ in the experimental test using NAS.

**Figure 14:**
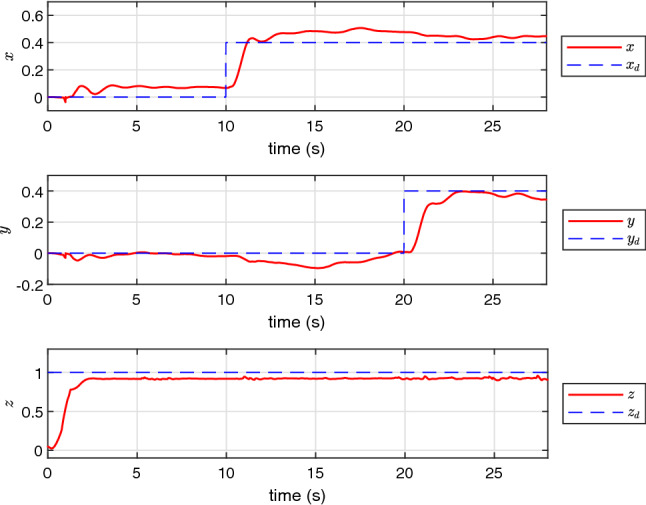
The profile of *x*, *y* and *z* in the experimental test using NAS.

**Figure 15:**
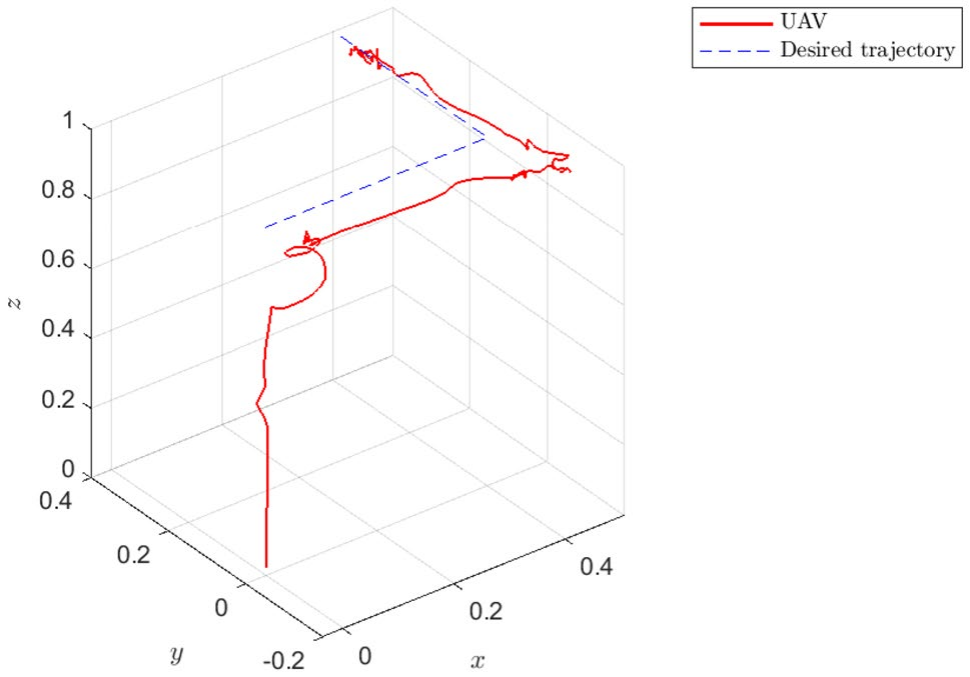
The profile of *x*, *y* and *z* in the experimental test using NAS in 3D.

Finally, the performance of NAS is also evaluated in a real scenario on an experimental UAV. The experimental results presented in Figs. 12, 13, 14 and 15 confirm that NAS is able to maintain the stable behaviour of the UAV and follow the desired trajectory closely in the experimental setting. More specifically, Figs. 12 and 13 illustrate the attitude rates of the UAV in the local coordinate system and the Euler rotational angles of the UAV in the global coordinate system respectively. Moreover, the profile of the translational motion of the UAV in the three dimensional space is illustrated in Figs. 14 and 15. To get a better view of the performance of the proposed adaptive algorithm in a real-life setting with parametric uncertainties, the fitness values for *x*, *y* and *z* in the steady state condition and from time 23*s* to 30*s* are calculated and listed in Table 6. The average fitness value in the table confirms that the controller performance is also good with real-life uncertainties. The transient response time under this setting can be seen in Table 7, where the rise and peak times of *x*,*y* and *z* are calculated from 10*s*, 20*s*, and 0*s*, respectively. Here, the rise time of each position state is the time required to reach $10\%$ and $90\%$ of the desired position.

**Table 3:** The fitness of rotational states of UAV.

Variable	CAS (%)	NAS (%)
*p*	99.7892	99.8793
*q*	99.7769	99.6634
*r*	98.7335	99.8990
Average	99.4332	99.8139

**Table 4:** The fitness of rotational states of UAV with $10\%$ of $\tau$.

Variable	CAS (%)	NAS (%)
*p*	30.7907	99.6707
*q*	37.5099	99.7074
*r*	48.2004	98.5925
Average	38.8337	99.3235

**Table 5:** The fitness of rotational states of UAV in the presence of multiple abrupt faults in $\tau$.

Variable	CAS (%)	NAS (%)
*p*	30.7479	99.6486
*q*	37.0523	99.7157
*r*	48.3842	96.8287
Average	38.7281	98.7310

**Table 6:** The fitness of the UAV translational states in the experimental case.

Variable	NAS (%)
*x*	91.0109
*y*	92.3043
*z*	91.7331
Average	91.6827

**Table 7:** Transient response time of the experimental setting.

Position	Rise time to reach $10\%$ target (s)	Rise time to reach $90\%$ target (s)	Peak time
*x*	0.001	1.08	1.47 s
*y*	0.555	2.53	No overshoot
*z*	0.5	2.08	No overshoot

## Conclusion and directions for future work

A fully tracking control for 6-DOF of UAV with unknown parameters is developed in this paper. A virtual PD controller is proposed to handle the tracking control of translational motions. Two adaptive schemes are designed and compared for rotational dynamics with the presence of unknown parameters in the nonlinear dynamics. The propeller inertia and translational drag are unknown for feedback control design. Also, an external disturbance containing an unknown constant is added to the dynamical system. CAS using the certainty equivalence principle is designed to handle the uncertain parameters. However, this scheme contains an inherent drawback, where the estimated parameters cannot be guaranteed to converge to the actual value of unknown parameters. To solve this issue, we develop NAS by adding a continuous function to the control structure. The adaptive law is able to guarantee to handle uncertain parameters with the appropriate design of the manifold. Moreover, this scheme has a simple structure and is more practical to be implemented. Several simulations are presented for a mini-drone to demonstrate the effectiveness of our scheme. We also conduct an experimental test to the real UAV using NAS to validate our design. It will be interesting to extend this scheme for multiple heterogeneous UAV with fully unknown time-varying parameters.

## Data Availability

The datasets generated and/or analyzed during the current study are not publicly available due to the confidentiality but are available from the corresponding author on reasonable request.
